# Needs-based considerations for the role of low-dose aspirin along the CV risk continuum

**DOI:** 10.1016/j.ajpc.2024.100675

**Published:** 2024-04-15

**Authors:** Francesca Santilli, Gerhard Albrecht, Michael Blaha, Angel Lanas, Li Li, Dirk Sibbing

**Affiliations:** a Department of Medicine and Aging and Center for Advanced Studies and Technology, University of Chieti, Chieti, Italy; b Medical & Clinical Affairs Consumer Health, Bayer U.S. L.L.C., Whippany, NJ, United States; c Johns Hopkins Ciccarone Center for the Prevention of Cardiovascular Disease, Baltimore, MD, USA; d University of Zaragoza, IIS Aragón, CIBERehd, Zaragoza, Spain; e Medical Affairs & Pharmacovigilance, Pharmaceuticals, Bayer AG, Berlin, Germany; f Ludwig-Maximilians University (LMU), Munich, Germany; g and Privatklinik Lauterbacher Mühle am Ostersee, Seeshaupt, Germany

**Keywords:** Aspirin, Primary prevention, Cardiovascular disease, Personalized treatment

## Abstract

Cardiovascular disease (CVD) remains the leading cause of death worldwide. The risk of a cardiovascular (CV) event is not static and increases along a continuum, making identification and management complex.

Aspirin has been the cornerstone of antiplatelet therapy in CV risk reduction and remains the only antiplatelet agent with current guideline recommendations throughout the CV risk continuum. In light of recent trials, the role of aspirin in CVD prevention in asymptomatic patients has been downgraded in clinical guidelines. However, a substantial proportion of asymptomatic patients have underlying conditions, such as advanced subclinical atherosclerosis that are associated with high CV risk. Advanced subclinical atherosclerosis has not been extensively investigated in patients in clinical trials but in the absence of significant bleeding risks, patients with subclinical atherosclerosis may particularly benefit from preventive aspirin therapy.

Recent studies and clinical guidelines support the need for a personalized treatment approach for these patients, balancing their risk of future CV events against their relative bleeding risk. In this commentary, we first discussed various tools and strategies currently available for assessing CV and bleeding risks; we then provided two hypothetical cases to outline how these tools can be implemented for optimal management of patients with no prior CV events who, nonetheless, are susceptible to CVD.

The first case details a young and apparently healthy patient with underlying advanced subclinical atherosclerosis; whereas the second case describes a patient with recently diagnosed type 2 diabetes mellitus who is at higher risk of CVD than their non-diabetic counterparts. For both cases, we considered patient clinical characteristics, CV and bleeding risks, as well as other risk factors to evaluate the appropriate treatment strategy and determine whether patients would obtain a net clinical benefit from low-dose aspirin therapy. These cases can serve as examples to guide clinical decision-making on the use of low-dose aspirin for primary CVD prevention and improve CVD management via a personalized approach.

## Highlights

• Recommendations for aspirin in patients with no apparent CV were downgraded in recent guidelines.
• Patients with advanced underlying subclinical atherosclerosis are at higher CV risk.
• A personalized approach balancing CV and bleeding risks identifies patients who may need aspirin.

## Graphical abstract

**Figure:**
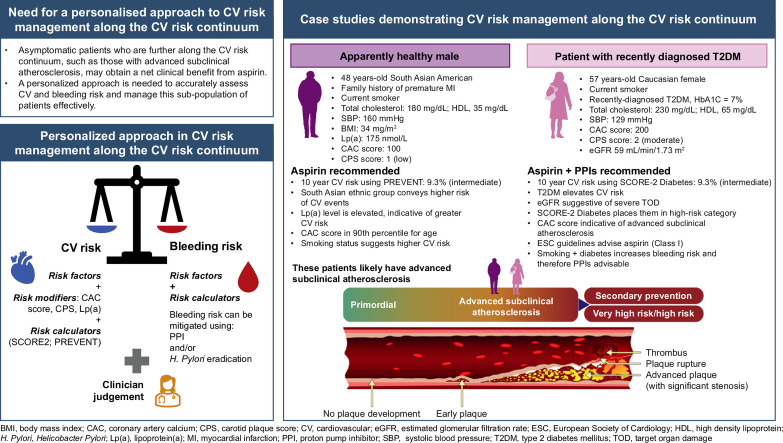

## 1 Introduction

Cardiovascular disease (CVD) is the leading cause of death globally [1]. The risk of cardiovascular (CV) events increases along a continuum, and patients may be at high risk both before and after an event, making it difficult to accurately assess a patient's CV risk [2]. Aspirin has been the cornerstone of preventive therapy and is the only antiplatelet agent currently recommended for use throughout the CV risk continuum. Despite the changing landscape of CV preventive therapies [3], major guidelines continue to recommend aspirin, both as monotherapy and combination therapy [4], for the prevention of CVD [5], including the management of chronic coronary syndrome (CCS) [6,7] and acute coronary syndrome (ACS) [8].

Aspirin remains an inexpensive and accessible drug, with its once-daily dosing regimen allowing better treatment adherence among patients relative to other antithrombotic agents that require twice-daily dosing [9].

The role of aspirin for patients without any previous CV events, traditionally referred to as “primary prevention”, has been downgraded as far as routine use in recent guidelines [5,10]. This was the result of the predominantly negative outcomes of three clinical trials – ASCEND (A Study of Cardiovascular Events in Diabetes) [11], ASPREE (Aspirin in Reducing Events in the Elderly) [12], and ARRIVE (Aspirin To Reduce Risk Of Initial Vascular Events) [13] – assessing aspirin in primary prevention. However, several limitations were evident in these trials. The ASCEND [11] trial demonstrated a statistically significant CV benefit for aspirin in patients with diabetes without evidence of CV disease, at the expense of an increase in major bleeding events. However, the ASCEND [11] included patients with well-controlled diabetes (on average), thus the absolute risk reduction with aspirin therapy might be even higher in subjects with poorly controlled disease. Moreover, only around a quarter of participants were prescribed proton-pump inhibitors (PPIs) to mitigate gastrointestinal (GI) bleeding [11]. The ARRIVE trial recruited a population with lower event rates than expected, making the study more representative of a low-risk population [12]. The ASPREE trial outcome was unusual among trials with aspirin in primary prevention in concluding that all-cause mortality was greater among patients receiving aspirin vs. placebo [13]. A secondary analysis of the ASPREE trial demonstrated a dramatic increase in net clinical benefit (NCB) of aspirin in those patients’ carriers of genetic variants associated with elevated Lp(a) [[14], [15]].

Studies by the United States Preventive Services Task Force (USPSTF) have shown that the absolute effect of decreasing CV events was higher (−2.5 % to −0.1 %) than that of increased bleeding (0.1 % to 1.0 %) with the use of low-dose aspirin among adults ≥40 years without known CVD [16]. They reported a net benefit of aspirin for both men and women at ≥5 % 10-year CVD risk levels when starting between age 40–59 years, and for those at ≥10 % 10-year CVD risk levels when starting between age 60–69 years [17]. Despite these favorable outcomes, the USPSTF issued a limited recommendation for aspirin use in primary prevention, stating that the decision to initiate aspirin therapy should be made individually by clinicians [18].

The 2019 American Heart Association/American College of Cardiology (AHA/ACC) guidelines assigned IIb recommendation for low-dose aspirin in high-risk primary prevention patients who are not at a high risk of bleeding [10]. Recent European Society of Cardiology (ESC) guidelines favor a personalized approach to CVD treatment which takes account of individual patient risk [5,19]. These recommendations emphasize the importance of determining the individual margin of net benefit among these patients.

It is, however, important to note that there is an asymptomatic sub-population of patients along the CV risk continuum, within the primary prevention category, who have underlying advanced subclinical atherosclerosis [2]. These patients are at a high risk of CV events and may benefit from aspirin therapy, particularly if they have a relatively low bleeding risk [20]. None of the three trials mentioned above [11], [12], [13] including ASCEND, enrolled patients according to the burden of subclinical atherosclerosis. This paper highlights some of the ways to assess CV and bleeding risks in these patients; shares recent data demonstrating the benefits of aspirin in patients with advanced subclinical atherosclerosis; and applies this approach using two hypothetical clinical cases.

## 2 Assessing cardiovascular and bleeding risks, and determining potential treatment benefits in asymptomatic patients with advanced subclinical atherosclerosis

The likely presence of advanced subclinical atherosclerosis may be determined through consideration of patient risk factors, risk modifiers (Suppl. Table 1), and the use of CV risk calculators (e.g., SCORE2, the pooled cohort equations [PCE], Predicting Risk of Cardiovascular Disease Events [PREVENT], Globorisk score, and QRISK3). It should be noted that risk calculators may overestimate or underestimate risks in certain groups such as young adults [21] and younger patients with metabolic syndrome en-route to type 2 diabetes mellitus (T2DM) [22], highlighting the importance of clinician judgment considering the aforementioned risk factors. The ESC recommends the estimation of 10-year fatal and non-fatal CV risk using the SCORE2 algorithm in apparently healthy people without established CVD, and the algorithm may be adjusted for competing risk factors in this population [5]. The SCORE2-Diabetes algorithm is specific to patients with T2DM and integrates conventional CVD risk factors along with diabetes-specific information, including HbA1c and estimated glomerular filtration rate (eGFR) [19]. The AHA/ACC guidelines recommend risk stratification using the PCE in asymptomatic patients to estimate 10-year CVD risk [10]. Recently the PCE has been updated to the PREVENT equations which also take into account other health conditions, such as kidney and metabolic diseases, for determining the 10- and 30-year chances of both atherosclerotic CVD [23].

**Table 1:** Clinical vignette of a hypothetical young, apparently healthy adult with advanced subclinical atherosclerosis.

Age	48 years old
Ethnicity	South Asian American male
Family history	Family history of premature MI
Smoking status	Current smoker
Total cholesterol	180 mg/dL
HDL Cholesterol	35 mg/dL
SBP	160 mmHg
BMI	34 kg/m²
eGFR	80 mL/min/1.73 m^2^
Lp(a) level	175 nmol/L
CAC score	100, 90th percentile
CPS	1 (low plaque burden)

BMI, body mass index; CAC, coronary artery calcium; CPS, carotid plaque score; HDL, high density lipoprotein; Lp(a), lipoprotein (a); MI, myocardial infarction; SBP, systolic blood pressure.

Coronary artery calcium (CAC) scoring is an especially useful tool in assessing atherosclerotic plaque burden and, therefore, estimating CV risk [24] The Multi-Ethnic Study of Atherosclerosis (MESA) demonstrated that CAC scoring can distinguish asymptomatic patients at higher risk of CVD but without a high risk of bleeding; though it should be noted that this was an observational study, with outcomes based on assumed relative risk reductions and relative risk increases for CV events and bleeding events, respectively [20]. Further analysis of MESA data showed that evaluation of subclinical atherosclerosis with CAC scoring significantly improved risk classification for patients with T2DM even 10 years after screening [25]. In addition, data from the CLARIFY registry study demonstrated that greater CAC burden was associated with increased risk of major adverse CV events in patients with T2DM, and that screening for CAC reduced CV risk [26]. Another prospective, population-based cohort study using participants from the Dallas Heart Study also reported a similar association between higher CAC score and increased CV risk in CVD-free patients [27].

The use of CAC scoring may also be useful in determining the potential treatment benefits of antiplatelet therapy. In asymptomatic patients with CAC scores ≥100 and ≥400, the number needed to treat (NNT) with aspirin to prevent CVD was lower than the number needed to harm (NNH) [20]. The National Lipid Association guidelines highlight that in the setting of low bleeding risk, a CAC score of ≥100 may distinguish a subgroup of patients in whom the benefits of low-dose aspirin therapy exceed its associated bleeding risk [28].

The ESC guidelines but not AHA/ACC guidelines, recommend carotid ultrasound to determine carotid plaque scores (CPS) as an alternative when CAC scoring on non-contrast computed tomography is not available or feasible. Elevated levels of lipoprotein (a) (Lp[a]) confer a higher risk of CVD and are encountered in almost 1 in 5 individuals [29]. Patients with genotypes associated with elevated Lp(a) levels in plasma have been shown to derive a greater NCB from aspirin [14,15].

Considerations for bleeding risk are necessary when deciding whether a patient should be prescribed antiplatelet therapy. A patient's bleeding risk is modulated by a variety of factors (see Suppl. Table 2); some of these overlap with CV risk factors while others influence CV risk independently. Notably, subclinical atherosclerosis is a CV risk enhancer that is independent of bleeding risk [28]. When making decisions on prescribing antiplatelet therapy, interactive bleeding risk calculators may be useful; examples of those specific to low-dose aspirin in primary prevention include the ASA-Risk Calculator (https://asarisk.doctime.es/) [30], Aspirin-Guide, and Health Navigator New Zealand [31].

Bleeding risk may be mitigated in patients who require antiplatelet therapy. PPIs, when combined with low-dose aspirin, are associated with an 80 % risk reduction in bleeding but are still underused for gastroprotection [32]. Their use in combination with aspirin is recommended by ESC guidelines [19]. Moreover, the HEAT trial demonstrated that eradication of the bacterium *H. Pylori* reduced peptic ulcer bleeding in older patients on aspirin for the first 2.5 years of primary prevention [33].

The following hypothetical case studies outline how the aforementioned tools can be implemented clinically to devise individual treatment strategies and assess potential benefits of low-dose aspirin.

## 3 Case study 1: subclinical atherosclerosis in a young, apparently healthy adult

The first clinical vignette describes an apparently healthy individual with premature advanced subclinical atherosclerosis (Table 1).

Increasing evidence suggests that a growing risk of CVD is present long before a CV event. Estimates suggest that around 50 % of men and 64 % of women who died of sudden cardiac death in the USA did not have a previous manifestation of CVD, and most of them were not considered high-risk according to the Framingham score [34]. Accordingly, ESC guidelines identify that apparently healthy people should be considered for CVD prevention [5]. While there is no risk score calculator specific to South Asian populations, the PREVENT risk calculator shows that this patient seems to have an intermediate estimated 10-year risk of CVD (9.3 %). Judging by this patient's ethnicity, family history of CVD, and CAC score, he is likely to have an even higher risk of CVD despite being apparently healthy. His-South Asian ethnicity, in particular, stands out as a risk factor since recent AHA/ACC guidelines have identified this ethnic group to be at high CV risk [35]. Research has also suggested South Asian men tend to have higher CAC burden than other ethnic groups [36].

As detailed earlier, the observed elevated Lp(a) levels is another CV risk factor, which in turn implies greater NCB from aspirin for this patient [14]. Given these clinical characteristics and high risks, the patient would likely benefit from low-dose aspirin therapy for CVD prevention.

## 4 Case study 2: patient with recently diagnosed T2DM

The second clinical vignette is of a patient with recently diagnosed T2DM (Table 2).

**Table 2:** Clinical vignette of a hypothetical patient with T2DM.

Age	57 years old
Ethnicity	Caucasian female
Smoking status	Current smoker
Diabetes	T2DM diagnosed 6 months ago
Hb1Ac	7 %
Total cholesterol	230 mg/dL
HDL Cholesterol	65 mg/dL
SBP	129 mmHg
CAC score	200, 90th percentile
CPS	2 (moderate plaque burden)
eGFR	59 mL/min/1.73 m^2^
Bleeding history	No prior history of gastrointestinal bleeding

CAC, coronary artery calcium; CPS, carotid plaque score; eGFR, estimated glomerular filtration rate; T2DM, type 2 diabetes mellitus.

Patients with T2DM are at a two-fold excess risk of CV events, independent of other risk factors [37]. Furthermore, patients with T2DM often have risk factors associated with CVD. The oxidative stress, platelet activation, increased coagulability, and endothelial dysfunction associated with T2DM may also contribute to CVD development [38]. The ESC guidelines recognize that patients with T2DM are at a higher risk of CV events, and those with severe target organ damage (TOD) (eGFR <45 ml/min/1.73m^2^, or eGFR 45–59 mL/min/1.73 m2 and microalbuminuria, or proteinuria, or presence of microvascular disease in at least three different sites) are regarded as at very high risk [5,19]. For these latter patients, assessment of SCORE2-Diabetes is not recommended and there is Class I recommendation for antithrombotic therapy, according to the ESC 2021 guidelines for cardiovascular prevention [5].

Applying the SCORE2-Diabetes CV risk calculator would place this patient in the “intermediate risk” category (10-year risk of a CV event 9.3 %).

Availability of albumin-to-creatinine ratio would have allowed evaluation for severe TOD. The presence of severe TOD would pose the patient in the very high-risk category, and no calculation of SCORE2-Diabetes is warranted. Based on the available data derived from ASCEND, aspirin in such patient with T2DM and no apparent CVD, with no further information about subclinical atherosclerosis, will likely reduce the incidence of a first serious vascular event by 12 %, at the expense of a 29 % increase in major bleeding [11]. Consistently, results from the ASCEND informed the guidelines with a IIb, class A recommendation for aspirin. Nevertheless, the high CAC score of >100 means that this patient falls into the group for whom the NNT with aspirin is lower than the NNH [20], which further substantiates the potential benefits from aspirin.

It is, however, equally important to recognize the increased bleeding risk associated with both aspirin use and other risk factors – including elevated eGFR and smoking – in this patient.

Strategies to mitigate potential bleeding risk associated with low-dose aspirin would be advisable for this patient based on the above observations. It has been suggested in the ASCEND trial that the absolute increase in GI bleeds may have been lower if PPIs were more frequently prescribed. Therefore, with all factors considered, we would recommend prescribing low-dose aspirin, preferably along with a PPI, for this patient to achieve primary CVD prevention while balancing bleeding risks.

## 5 Conclusions and future directions

After 125 years, aspirin continues to be the antithrombotic of choice in the prevention of CVD before an event. Recent large-scale studies have demonstrated that traditional risk assessment and the binary classification into “primary” and “secondary” prevention may fail to identify a significant subset of high-risk patients who would likely benefit from aspirin. Patients with a higher CV risk and lower bleeding risk, including those with conditions described in the above cases, would benefit from a personalized approach to CVD prevention. Future randomized clinical trials should enroll patients according to CAC score and/or carotid ultrasound or at least provide more detailed information about subclinical atherosclerosis, that is currently lacking even in the majority of trials including the ASCEND. Clinician education is warranted to improve decision-making when determining patients at high CV risk who would benefit from aspirin therapy.

## Disclosure

FS, MB, AL, and DS are members of the Bayer AG global advisory board. LL and GA are employees of Bayer, AG, Berlin, Germany.

## Funding

No funding was provided for the development of this manuscript

## CRediT authorship contribution statement

**Francesca Santilli:** Writing – review & editing, Writing – original draft, Data curation, Conceptualization. **Gerhard Albrecht:** Writing – review & editing, Writing – original draft, Data curation, Conceptualization. **Michael Blaha:** Writing – review & editing, Writing – original draft, Data curation, Conceptualization. **Angel Lanas:** Writing – review & editing, Writing – original draft, Data curation, Conceptualization. **Li Li:** Writing – review & editing, Writing – original draft, Data curation, Conceptualization. **Dirk Sibbing:** Writing – review & editing, Writing – original draft, Data curation, Conceptualization.

## Declaration of competing interest

The authors declare the following financial interests/personal relationships which may be considered as potential competing interests:

Prof Francesca Santilli reports a relationship with Bayer AG that includes: consulting or advisory and speaking and lecture fees. Prof Dirk Sibbing reports a relationship with Bayer AG that includes: consulting or advisory and speaking and lecture fees. Dr Michael Blaha reports a relationship with Bayer AG that includes: consulting or advisory. Prof Angel Lanas reports a relationship with Bayer AG that includes: consulting or advisory. Gerhard Albrecht reports a relationship with Bayer AG that includes: employment. Li Li reports a relationship with Bayer AG that includes: employment. If there are other authors, they declare that they have no known competing financial interests or personal relationships that could have appeared to influence the work reported in this paper.

## Appendix Supplementary materials

Image, application 1
