# Supplementary material for: Needs-based considerations for the role of low-dose aspirin along the CV risk continuum
Source: Am J Prev Cardiol. 2024 Apr 15;18:100675. doi: 10.1016/j.ajpc.2024.100675 (PMC11061695; doi:10.1016/j.ajpc.2024.100675)
Supplement: Supplementary file 1 [file mmc1.docx]

## Supplementary information

| CVD Risk | |
| --- | --- |
| Risk factors | **Risk modifiers** |
| Age | Ethnicity/geography |
| Sex | Family history |
| Ethnicity | Ankle Brachial Index (ABI) score |
| Alcohol consumption | Alcohol consumption |
| BP/hypertension | Apolipoprotein B (ApoB) level |
| BMI/obesity | CAC score |
| Diabetes | Carotid artery US |
| Diet | hs-CRP test outcome |
| Dyslipidemia | Lp(a) level |
| Physical activity |  |
| Smoking |  |

Suppl. Table 1. Risk factors & risk modifiers for CVD and risk factors for bleeding. BMI, body mass index; BP, blood pressure; CAC, coronary artery calcium; CVD, cardiovascular disease; hs-CRP, high-sensitivity C-reactive protein; Lp(a), lipoprotein (a); PPI, proton pump inhibitor; US, ultrasound.

| Bleeding Risk |
| --- |
| Risk factors |
| Age  Sex  Smoking  Diabetes  BMI/obesity  Alcohol consumption  BP/hypertension  Concomitant medications  Comorbid conditions  Dyspepsia & peptic ulcers  *H. Pylori* infection  PPI/gastroprotective strategy |

Suppl. Table 2. Risk factors risk factors for bleeding. BP, blood pressure, PPI, proton pump inhibitor
